# Recent advances in understanding neuropathic pain: glia, sex differences, and epigenetics

**DOI:** 10.12688/f1000research.9621.1

**Published:** 2016-11-22

**Authors:** Halina Machelska, Melih Ö. Celik

**Affiliations:** 1 Department of Anesthesiology and Critical Care Medicine, Charité-Universitätsmedizin Berlin, Campus Benjamin Franklin, Berlin, Germany

**Keywords:** neuropathic pain, glial cells, microglia, astrocytes, oligodendrocytes

## Abstract

Neuropathic pain results from diseases or trauma affecting the nervous system. This pain can be devastating and is poorly controlled. The pathophysiology is complex, and it is essential to understand the underlying mechanisms in order to identify the relevant targets for therapeutic intervention. In this article, we focus on the recent research investigating neuro-immune communication and epigenetic processes, which gain particular attention in the context of neuropathic pain. Specifically, we analyze the role of glial cells, including microglia, astrocytes, and oligodendrocytes, in the modulation of the central nervous system inflammation triggered by neuropathy. Considering epigenetics, we address DNA methylation, histone modifications, and the non-coding RNAs in the regulation of ion channels, G-protein-coupled receptors, and transmitters following neuronal damage. The goal was not only to highlight the emerging concepts but also to discuss controversies, methodological complications, and intriguing opinions.

## Introduction

Neuropathic pain is a severe chronic disease that can develop following lesions to the central nervous system (CNS) (spinal cord injury, stroke, and multiple sclerosis) or to peripheral nerves. The peripheral nerve damage can be caused by diseases (diabetes and herpes zoster) or trauma (compression, stretch, and amputation) ^1^. Patients experience spontaneous burning or shooting pain, which can be amplified by mechanical and thermal stimuli, both noxious (pressure, heat, and cold) and normally innocuous (touch and warm or cool temperatures) ^2^. Given that current therapies are unsatisfactory ^3^, the investigation of mechanisms by which nerve damage triggers pain remains the focus of research, the goal of which is to find new targets for better treatment. The neuropathic pain pathophysiology is complex and includes peripheral and central neuronal alterations ^4– 6^ as well as neuro-immune interactions ^7– 11^. In this article, we focus on the findings of the last 3 to 4 years in the very dynamic and expanding areas of epigenetics and glia, immune cells of the CNS, including sex differences in neuropathic pain.

## Glia in the modulation of neuropathic pain

It has been established that neuropathic pain is not exclusively a sensory phenomenon but also involves immune reactions. Glia represent immune cells of the CNS; microglia and astrocytes are the most investigated ^9– 11^, and oligodendrocytes have emerged as new players relevant to neuropathic pain.

## Microglia

**Figure 1.**
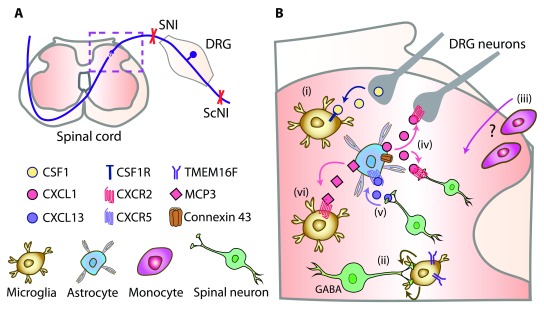
Novel neuro-glial mechanisms of neuropathic pain. ( **A**) Schematic innervation of the dorsal horn spinal cord (dashed box) by DRG neurons. Neuropathy was induced by SNI or ScNI in mice or rats. ( **B**) Neuro-glial mechanisms in the dorsal horn spinal cord (dashed box in A): (i) CSF1 released from injured DRG neurons activates CSF1 receptor (CSF1R) in microglia ^15, 16^. (ii) TMEM16F in microglia mediates their phagocytosis of GABAergic-interneuron terminals ^17^. (iii) Whether monocytes infiltrate spinal cord and their relative role versus resident microglia are still unclear ^22, 26, 28, 29, 32^. (iv) Upregulated connexin 43 in astrocytes triggers the secretion of CXCL1 to activate CXCR2 on DRG neuron central terminals and spinal interneurons ^33^. (v) Spinal neuron-derived CXCL13 activates CXCR5 in astrocytes ^34^. (vi) MCP-3 secreted from astrocytes activates CXCR2 in microglia ^74^. CSF1, colony-stimulating factor 1; CXCR, chemokine CXC receptor; DRG, dorsal root ganglion; GABA, γ-aminobutyric acid; MCP-3, monocyte chemotactic protein-3; ScNI, sciatic nerve injury; SNI, spinal nerve injury.

Following peripheral nerve injury, microglia proliferate, become hypertrophic and activated, and secrete molecules which sensitize sensory neurons in the spinal cord. The best-characterized mechanism involves the activation of microglial purinergic receptors P2X4 by adenosine triphosphate (ATP) secreted from damaged neurons or astrocytes or both. This stimulates microglia to release brain-derived neurotrophic factor (BDNF), which activates its neuronal receptors. Ultimately, inhibitory signaling mediated by γ-aminobutyric acid A (GABA _A_) receptors is diminished and this culminates in enhanced pain ^11– 14^. The microglia–neuronal interactions are bidirectional, and there is some dispute of the initial signal that activates microglia. In addition to the ATP/P2X4 pathway, the dorsal root ganglion (DRG) neuron central endings (which terminate in the spinal cord dorsal horn) ( Figure 1A) secrete chemokines such as CX3CL1, CCL2, and CCL21, which activate their respective receptors on microglia ^13^. The previous concepts have recently been challenged by studies suggesting that it is a cytokine colony-stimulating factor 1 (CSF1) which is upregulated in injured DRG neurons, transported, and secreted in the spinal cord, where it activates CSF1 receptors in microglia, leading to their proliferation and neuropathic pain induction ^15, 16^ ( Figure 1Bi). Selective neuronal CSF1 deletion reduced microglia proliferation and prevented neuropathy-triggered hypersensitivity in mice. Downstream of CSF1 receptor, the microglial membrane adaptor protein DAP12 was required for hypersensitivity but surprisingly not for microglia proliferation. Hence, CSF1-mediated hypersensitivity engaged DAP12-dependent upregulation of pain-related genes (including the gene encoding P2X4 receptor) and DAP12-independent microglia proliferation ^15^.

As phagocytic cells, microglia remove apoptotic tissue following injury. New work proposes that a Ca ^2+^-dependent channel, TMEM16F, in microglia mediates their phagocytic activity, which leads to the shortening of GABAergic interneuron terminals, subsequent loss of GABA-mediated inhibitory transmission, and neuropathic pain ^17^ ( Figure 1Bii). The concept is in line with recently postulated loss of synaptic contacts between spinal parvalbumin-expressing inhibitory interneurons and excitatory interneurons leading to their disinhibition ^18^, and this represents an alternative mechanism to the controversial apoptosis of inhibitory interneurons ^19, 20^. Selective depletion of microglial TMEM16F diminished microglia-mediated phagocytosis, preserved neuronal GABA immunoreactivity, and abolished hypersensitivity following sciatic nerve injury (ScNI) in mice. Thus, microglia phagocytic activity may contribute to neuropathic pain ^17^.

**Figure 2.**
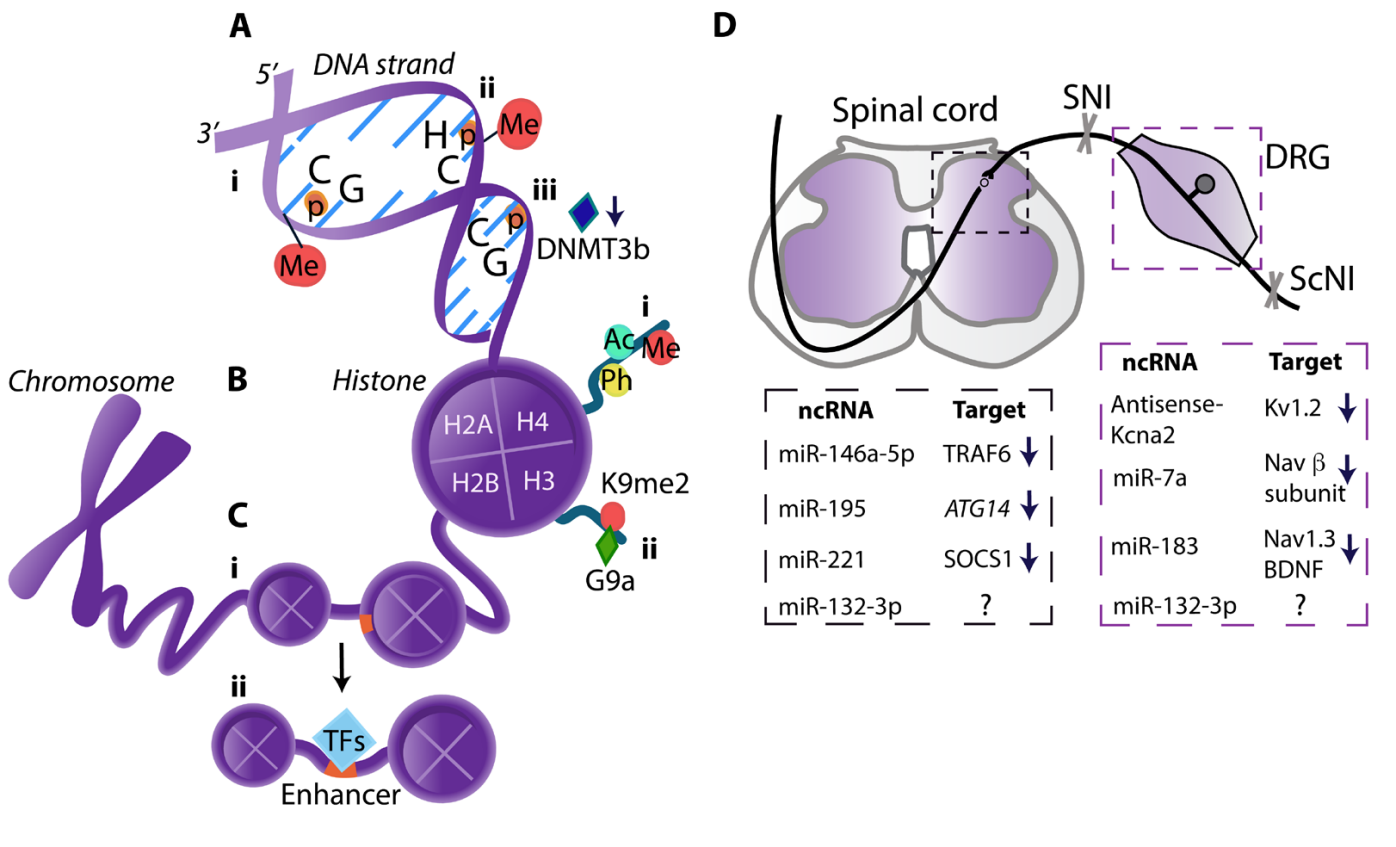
Epigenetic modifications in neuropathic pain. ( **A**) DNA methylation (Me) occurs mostly at the cytosine of CpG sites ^55, 61^ (i) but can also happen at the cytosine of CpH sites (H = A, T, or C) ^67^ (ii). (iii) DNA methyltransferase 3b (DNMT3b) downregulation leads to the demethylation at the CpG sites in the purinergic P2X3 receptor gene promoter ^70^. ( **B**) Histone modifications: (i) DNA is wrapped around histone octamers that consist of histones H2A, H2B, H3, and H4. Histone tails, particularly on H3 and H4, can be acetylated (Ac), phosphorylated (Ph), or Me; (ii) Dimethyltransferase G9a mediates dimethylation (me2) of lysine 9 (K9me2) on histone H3 of the promoters of genes encoding K ^+^ channels ^72^ or MOR ^73^. ( **C**) Enhancers (orange) are non-coding DNA regions that can be activated by local nucleosome remodeling and binding of transcription factors (TFs), thereby influencing gene expression ^21^: (i) An inactive enhancer is packed in compact chromatin and cannot be bound by TFs. (ii) Following nerve injury, increased TF binding at spinal microglia enhancers was identified for genes encoding complement system member C4b and chemokines CCL5 and CCL12 [22]. ( **D**) ncRNAs and their targets in the spinal cord and DRG (listed in the respective dashed boxes) involved in neuropathic pain following SNI or ScNI in mice or rats. ncRNAs include analgesic miRNAs (miR-7a, miR-183, miR-146a-5p) ^80– 82^ and pain-promoting long non-coding RNA (Kcna2 antisense RNA) ^79^ or miRNAs (miR-195, miR-221, miR-132-3p) ^83– 85^. ↓, downregulation; ↑, upregulation; ?, unidentified target; *ATG14*, gene encoding protein that facilitates autophagy; BDNF, brain-derived neurotrophic factor; CpG, cytosine–phosphate–guanine dinucleotide; DRG, dorsal root ganglion; Kv, voltage-gated potassium channel; miRNA, microRNA; MOR, µ-opioid receptor; Nav, voltage-gated sodium channel; ncRNA, non-coding RNA; ScNI, sciatic nerve injury; SNI, spinal nerve injury; SOCS1, suppressor of cytokine signaling 1, a protein suppressing cytokine signaling; TRAF6, tumor necrosis factor (TNF) receptor associated factor 6, an adapter protein mediating TNF-α signaling.

Mechanisms underlying neuropathic pain chronification gain increasing attention. This was recently addressed by analyzing latent enhancers. Enhancers are DNA regulatory regions that bind transcription factors to influence gene expression ^21^. Following ScNI in mice, the increased binding at enhancers close to genes encoding complement system member C4b and chemokines CCL5 and CCL12 was identified in spinal microglia ( Figure 2C). Since these effects lasted for a relatively long time (4 weeks) after the injury, when most transcriptional changes returned to normal, the enhancer profile alterations may account for pain persistency ^22^. Intriguingly, the study made several observations contradicting previous findings, including the absence of the gene encoding BDNF considered a crucial microglia–neuronal signaling molecule ^13, 14, 23^ (see also above). Furthermore, in contrast to previous data ^24– 27^, there was no spinal cord infiltration by blood-derived monocytes/macrophages and T lymphocytes, and thus resident microglia were regarded as the major spinal immune cells (99%) orchestrating neuropathic pain. Suggested reasons for discrepancies include the use of unspecific cell markers in previous studies versus microglia-specific purinergic receptor P2Y12 antibody ^22^.

The exclusive role of resident spinal microglia was explored by Tashima *et al.*, who proposed that irradiation and bone marrow transplantation, often employed in earlier studies, possibly impair the blood–spinal cord barrier, which represents an experimental artifact underlying monocyte/macrophage extravasation in the spinal cord ^28^. This was supported by the absence of spinal cord infiltration by circulating monocytes by using less toxic irradiation protocols, parabiotic mice, and mice with genetically labeled microglia. Also, Gu *et al.* postulated preferential involvement of resident microglia by employing P2Y12 immunostaining and double transgenic mice in which microglia and monocytes were differently labeled ^29^. Importantly, inhibition of microgliosis by an anti-mitotic drug diminished hypersensitivity following spinal nerve injury (SNI). However, the emphasis on microglia proliferation contradicts findings on the dissociation between microgliosis and pain ^30, 31^. Indeed, the upregulation of purinergic receptors and BDNF, and p38 mitogen-activated protein kinase phosphorylation in spinal microglia, but not their morphological alterations, are considered critical for neuropathic pain ^27^. Nevertheless, despite proposing the sole role of resident microglia ^29^, the concurrent study of this group claims the synergistic action of microglia and monocytes ^32^. Depletion of chemokine CX3C receptor (R) 1-expressing cells (including spinal microglia and peripheral monocytes/macrophages) attenuated SNI-induced hypersensitivity in mice. Postulated selective microglia depletion (using diphtheria toxin receptor transgenic mice) produced only transient analgesia. However, this transgenic approach caused compensatory elevations in astrocyte numbers, which could account for the re-emergence of hypersensitivity. Additionally, clodronate liposome-induced depletion of blood monocytes did not attenuate hypersensitivity, suggesting that they were not involved; monocytes were not detected in the spinal cord either. Furthermore, liposome treatment caused a compensatory increase in a circulating mononuclear cell subset ^32^. Together, these studies yielded conflicting findings, and the relative contribution of resident microglia versus astrocytes and infiltrating monocytes ( Figure 1Biii) and the relevance of microglia proliferation to neuropathic pain remain inconclusive.

## Astrocytes

Following peripheral nerve damage, spinal astrocytes proliferate and produce pro-inflammatory cytokines (for example, interleukin-1β [IL-1β]), matrix metalloproteinases, and chemokines CCL2, CCL7, and CXCL1 ^27, 30^. An additional mechanism involves connexin 43, a protein which forms gap junctions and exerts hemichannel activity. Thus, (presumably) microglia-derived tumor necrosis factor-α (TNF-α) selectively upregulated connexin 43 in astrocytes and triggered the secretion of CXCL1 to activate CXCR2 on neurons ( Figure 1Biv), which resulted in hypersensitivity following ScNI in mice. Inhibition of this pathway, including astrocyte depletion, attenuated the hypersensitivity. Since these effects occurred at later neuropathy stages, targeting astrocytic connexin 43 may be therapeutically more promising than interfering with early, microglia-mediated responses ^33^. In addition to astroglial–neuronal interactions ^33^, novel neuronal–astroglial communication has been proposed; it involves neuron-derived chemokine CXCL13, which activates CXCR5 in astrocytes ^34^ ( Figure 1Bv). Blocking the CXCL13/CXCR5 pathway suppressed SNI-induced hypersensitivity in mice. Nevertheless, the neuronal–astrocytic interaction involving CXCL13/CXCR5 will need confirmation since CXCL13 was also found in microglia and macrophages. Furthermore, CXCR5 was also expressed in spinal neurons ^34^ and thus direct neuronal CXCR5 activation, without astrocytic contribution, cannot be excluded.

Astrocytes are not electrically excitable, but they induce metabotropic glutamate receptor (mGluR)-mediated Ca ^2+^ oscillations and synaptogenic thrombospondin 1 (TSP-1) release, which are involved in neuronal circuit formation during development but typically are downregulated in adulthood. New work elegantly shows that the ScNI resulted in the re-emergence of mGluR5 signaling in cortical astrocytes in the adult mouse brain. Nerve injury enhanced levels of neuron-derived glutamate, which activated mGluR5 in astrocytes in the cortex. This elicited Ca ^2+^ transients and the release of astrocytic TSP-1, which in turn activated neuronal α2δ1 receptors to induce new synapse formation. Blocking astrocytic Ca ^2+^ elevation or synaptic formation (using α2δ1 blocker gabapentin) suppressed neuropathy-triggered hypersensitivity ^35^. These findings are interesting since gabapentin is used clinically. However, as some patients benefit from the treatment, the analgesic effects are moderate and side effects (dizziness, somnolence) may lead to therapy termination ^36– 38^. Hence, indications of novel therapeutic targets resulting from this research still need to be defined.

## Oligodendrocytes

Classically, oligodendrocytes produce myelin for axonal insulation in the CNS, and their role in pain modulation is just emerging. Genetic oligodendrocyte ablation in naïve mice (employing a diphtheria toxin receptor system) induced mechanical and cold but not heat hypersensitivity. This was associated with axonal degeneration and swelling (but not neuronal loss) in the spinal cord and was independent of demyelination, lymphocyte infiltration, and gliosis. In summary, oligodendrocyte loss induced symptoms of central neuropathic pain, supporting their requirement for normal somatosensation ^39^.

On the other hand, oligodendrocytes may contribute to the generation of neuropathic pain by secretion of IL-33, which can activate its receptors in microglia and astrocytes (and possibly neurons), leading to the release of other pro-inflammatory cytokines (TNF-α and IL-1β). This has been suggested in a mouse ScNI model and is based mostly on the IL-33 immunohistochemical detection in oligodendrocytes; however, the direct contribution of oligodendrocytes to hypersensitivity was not demonstrated ^40^.

## Glia and sex differences

Pain-related sex differences are receiving increased attention ^41– 43^. An intriguing recent suggestion is that microglia play a role in males only but that in females T lymphocytes infiltrating the spinal cord mediate neuropathic pain ^44^. This was supported by the reversal of ScNI-induced hypersensitivity by glia inhibitors (minocycline, fluorocitrate, and propentofylline) in male, but not female, mice. Hence, the classic pathway involving P2X4–p38–BDNF and disinhibition of spinal GABA _A_-mediated transmission contributed to neuropathic pain in males only. In females, T lymphocytes expressing peroxisome proliferator-activated receptor-γ (PPAR-γ) drove the hypersensitivity. Importantly, microglial hypertrophy and proliferation did not differ between sexes ^31, 44^. The male-specific p38 activation and signaling in spinal microglia, independent of proliferation, were also described in mice and rats following nerve damage in another study ^45^. These sex-related differences are suggested to be spinal cord specific. Attenuation of neuropathy-induced hypersensitivity in female mice following systemic minocycline ^46^ or in both sexes after systemic p38 inhibitor ^45^ was attributed to actions on peripheral immune cells ^45, 47^.

Nevertheless, other authors found neuropathy-induced upregulation of microglia and astrocytes expressing phosphorylated p38 also in female mice ^48^, indicating that spinal p38 activation is not always male specific ^45^. Additionally, spinally administered PPAR-γ agonist attenuated hypersensitivity in male rats ^49^, contrasting the predominant role of PPAR-γ in female mice ^44^. Furthermore, other studies found no differences between female and male mice in microglia-mediated hypersensitivity ^17, 32^. Future research will need to clarify whether the sex-related role of glia is restricted to selected mechanisms or models of neuropathic pain and how these findings are relevant to clinical pain. Clearly, there is increasing pressure to consider sex in preclinical research. Whereas many scientists are concerned, this will double the number of animals used, an intriguing opinion claims that this is unjustified since “there is no need to use enough animals to ensure that there is sufficient statistical power to detect all quantitative sex differences” ^50^. Although statistical significance should not be overestimated, the quality of statistical analysis is a strong focus of current recommendations ^51– 54^ and in many cases is strictly required. For example, according to German regulations, the sample size estimation and statistical power analysis are prerequisites for study approval by ethics committees.

## Epigenetic modifications and neuropathic pain

Epigenetic modifications, which include DNA methylation, histone modifications, and expression of non-coding RNAs (ncRNAs), lead to changes in gene expression but do not alter the actual DNA sequence. They are induced by stress, tissue damage, and disease and can affect both physiological and pathological processes ^55– 57^. During pain progression, the activity of genes encoding ion channels, receptors, neurotransmitters, and modulators is enhanced or suppressed abnormally ^58^. Here, we address recent findings on epigenetic modifications along the neuro-immune pain pathways.

## DNA methylation

DNA is mostly methylated at the cytosine of cytosine–phosphate–guanine (CpG) dinucleotide ( Figure 2Ai). CpG sites are distributed throughout the DNA sequence, including promoters, gene bodies, and transposable elements ^55, 59^. DNA methylation is tissue/cell type specific and forms differentially methylated regions (DMRs), which reflect tissue-specific function ^60^. Epigenome analysis of monozygotic twins who have almost identical genetic material but different methylation profiles might help in revealing DMRs associated with low or high pain sensitivity. Genome-wide DNA methylation analysis in blood leukocytes revealed that individuals with lower heat pain thresholds had increased DNA methylation in the promoter of the gene encoding transient receptor potential ankyrin 1 (TRPA1). In skin biopsies, the TRPA1 gene was upregulated in individuals with higher pain thresholds ^61^. Similarly, burning pain sensation was correlated with TRPA1 gene hypermethylation, and the hypermethylation was associated with TRPA1 mRNA downregulation in blood cells of a mixed group of patients with back pain and post-herpetic neuralgia ^62^. These findings are intriguing since, in mammals, nociceptor TRPA1 senses noxious stimuli and may be activated by cold but not heat ^63, 64^. Interestingly, another study found that high DMR numbers (72%) overlapped between blood T lymphocytes and prefrontal cortex following ScNI in rats ^65^. This suggests the feasibility of DNA methylation changes in T lymphocytes as non-invasive biomarkers of neuropathic pain. Nevertheless, only 43% of the promoters were altered in the same direction in both tissues ^65^ and thus careful tissue/cell type-specific analysis of methylation patterns is needed to adequately correlate them with pain intensity. Additionally, precise DNA sequence assessment may be important; for example, methylated CpGs are also located in intergenic regions, where they have no effect on gene activity. Moreover, although CpG methylation in the promoters typically represses transcription, methylation in the gene bodies can increase mRNA expression ^66^. Though not yet addressed in pain conditions, methylation also occurs at the CpH sites (H = A, T, or C) ( Figure 2 Aii) and was shown to be the dominant form of DNA methylation in human and mouse frontal cortex neurons ^67^. Notably, recent technical advances enable the generation of high-resolution epigenetic libraries and precise epigenetic analysis ^68, 69^.

Pain can also be associated with DNA demethylation. Demethylation in the purinergic P2X3 receptor gene promoter ( Figure 2Aiii) and its binding of transcription factor p65 resulted in P2X3 receptor protein upregulation in the DRG and diabetic neuropathic pain in rats. As these data are mechanistically interesting, their therapeutic relevance seems less clear since interfering with this pathway by blocking p65 appeared to be less effective than the P2X3 receptor antagonist treatment in producing analgesia ^70^.

## Histone modifications

Genomic DNA is wrapped around histone proteins, which can be post-translationally modified by methylation, acetylation, or phosphorylation ^56^ ( Figure 2Bi). Generally, histone acetylation and phosphorylation increase transcription whereas histone methylation can either silence or increase gene expression, depending on the methylated lysine ^71^. Laumet *et al.* ^72^ examined histone methylation at genes encoding voltage-gated (Kv1.4, Kv4.2, and Kv7.2) and Ca ^2+^-activated K ^+^ channels; opening of these channels results in decreased neuronal excitability. Following SNI in rats, while DNA methylation was unaltered, the dimethyltransferase G9a-mediated lysine 9 dimethylation on histone H3 (H3K9me2) of the promoters of genes encoding K ^+^ channels in the DRG was increased ( Figure 2Bii), resulting in the channels’ downregulation and correlating with hypersensitivity. Consistently, the G9a inhibition normalized the expression of K ^+^ channels and attenuated hypersensitivity ^72^. The G9a was also shown to methylate the promoter of the gene encoding µ-opioid receptor (MOR), which is activated by opioids such as morphine. SNI-induced downregulation of MOR gene and protein was associated with highly enriched levels of G9a product H3K9me2 in the MOR gene promoter. The G9a blockers restored MOR expression and enhanced morphine-induced analgesia. Additionally, in mice with G9a conditional knockout in the DRG neurons, neuropathy did not reduce MOR levels and morphine-induced analgesia ^73^. In summary, the G9a can silence several K ^+^ channels and MOR on the transcriptional level. Blocking G9a activity may improve the efficacy of opioid analgesics and prevent the chronification of neuropathic pain.

Histone modifications can also influence astrocyte–microglia interactions. ScNI in mice elevated the expression of pro-inflammatory cytokine monocyte chemotactic protein-3 (MCP-3) (also known as chemokine CCL7) exclusively in spinal astrocytes ( Figure 1Bvi). This was associated with the decreased histone methylation on MCP-3 promoter. MCP-3 application activated microglia, which was reversed by MCP-3 antibody, resulting in diminished neuropathy-induced hypersensitivity. Thus, MCP-3 promoter histone hypomethylation-mediated microglia activation by MCP-3 secreted from astrocytes in the spinal cord may represent a new target for neuropathic pain treatment ^74^.

## Non-coding RNAs

ncRNAs are RNAs that do not encode proteins. They are classified according to their size and function, and the major members include microRNAs (miRNAs) and long ncRNAs (lncRNAs). miRNAs contain 20 to 24 nucleotides and post-transcriptionally suppress protein expression by mRNA degradation or inhibition of translation. lncRNAs are longer than 200 nucleotides and can interfere with transcription and post-transcriptional processes by interacting with DNA, RNA, or protein ^56, 75^. There is abundant evidence on pain modulation by miRNAs, but the role for lncRNAs is much less known ^76– 78^. Below, we discuss recent research exploring the targets and functional relevance of ncRNAs in neuropathy.

A single study examining lncRNAs in neuropathic pain found that increased expression of a lncRNA named Kcna2 antisense RNA (as it is complementary to Kcna2 mRNA which encodes the Kv1.2 channel) led to Kcna2 mRNA downregulation in DRG neurons following SNI in rats ( Figure 2D). Blocking this lncRNA restored Kcna2 expression and attenuated SNI-triggered hypersensitivity ^79^.

There are more data on the role of both analgesic and pain-inducing miRNAs ( Figure 2D). miR-7a suppressed DRG neuron excitability by targeting the β2 subunit of voltage-gated Na ^+^ (Nav) channels after SNI in rats. SNI-induced miR-7a downregulation was associated with the β2 subunit protein upregulation in injured DRG. Locally injected virus expressing miR-7a diminished β2 subunit expression and alleviated hypersensitivity ^80^. miR-183 exerted analgesia by targeting Nav1.3 and BDNF in DRG. SNI-induced miR-183 downregulation was restored by spinal viral miR-183 delivery, resulting in decreased expression of Nav1.3 and BDNF mRNAs in DRG and diminished hypersensitivity in rats ^81^. Also, spinally applied miR-146a-5p mimic (a synthetic double-stranded RNA that mimics endogenous miR-146a-5p) reduced SNI-induced hypersensitivity in mice, probably by suppressing TNF receptor-associated factor 6, an adapter protein mediating signaling of TNF-α ^82^. Together, these studies indicate the restoration of analgesic miR-7a, miR-183, and miR-146a-5p as potential neuropathic pain treatments.

Alternatively, beneficial effects may be achieved by inhibition of pain-inducing miRNAs ( Figure 2D). miR-195 was upregulated in the spinal cord microglia, which was associated with decreased microglial autophagy (self-degradation) and enhanced SNI-induced hypersensitivity in rats. Consistently, spinal miR-195 inhibition enhanced microglial autophagy and decreased hypersensitivity. The miR-195-mediated actions were suggested to result from targeting gene *ATG14* which regulates autophagy, since miR-195 overexpression downregulated *ATG14* and inhibited autophagy. Thus, promoting microglia autophagy via miR-195 inhibition may attenuate neuropathic pain ^83^. Analgesia was also produced by inhibition of miR-221, which resulted from the upregulation of suppressor of cytokine signaling 1, a protein suppressing cytokine signaling. miR-221 was upregulated in the spinal cord microglia following ScNI. Spinal application of miR-221 inhibitor downregulated pro-inflammatory cytokines and diminished neuropathy-induced hypersensitivity ^84^. Pain-promoting actions of miR-132-3p were addressed in both neuropathic pain patients and rats. miR-132-3p was upregulated in blood leukocytes and peripheral nerve biopsies of neuropathic pain patients. A similar effect was found in the spinal cord and DRG (but not nerve) in rats with ScNI, and spinal miR-132-3p antagonism reversed hypersensitivity. However, the miR-132-3p target responsible for its pain-promoting effects remains elusive since, in contrast to the expected upregulation, it downregulated the expression of the glutamate receptor ^85^.

## Open questions, opportunities, and challenges

Recent research resulted in interesting novel findings that considerably advance our understanding of neuropathic pain pathogenesis and offer numerous new intervention targets, including cytokines (CSF1) ^15, 16^; TMEM16F-mediated microglial phagocytosis ^17^; microglial enhancers ^22^; astrocytic connexin 43, CXCL13/CXCR5, or cortical synaptic plasticity ^33– 35^; oligodendrocyte-based signaling ^40^; G9a-mediated histone methylation ^72, 73^; and many ncRNAs ^79– 85^. The key questions are which of these effects will be replicated and tested in patients and will any of them prove clinically effective. Elaborated transgenic approaches help to address specific cell type or signaling pathways but may result in compensatory changes. This is particularly true when manipulating the immune system ^32, 86^, whose role is to rapidly react to alterations in the body. Not surprisingly, several controversies emerged ^22, 29, 32^, and they certainly will stimulate further research. Additional remaining questions include the relative role of different glia types and of resident versus circulating leukocytes infiltrating the CNS, exploration and promotion of protective effects of glia, understanding human glia pathology, and the precise identification of epigenetic mechanisms. Given that chronic pain is mediated by a plethora of functionally redounding mediators and signaling pathways, the ability of a single epigenetic tool to modify multiple gene transcripts offers a possibility to target multiple pain mediators simultaneously and this has higher potential for successful pain treatment compared with targeting single molecules. On the other hand, this multimodal feature of epigenetic mechanisms carries risks of compensatory changes and side effects. An additional challenge is to achieve sufficient and tissue-relevant delivery of epigenetic modulators without inducing off-target actions.

Previous clinical trials, including those which tested glial modulators, have failed to produce sufficient pain relief in patients with neuropathic conditions ^13, 30, 87^. It is essential to acknowledge that chronic pain is a biopsychosocial experience and that not only biologic but also psychological and social factors result in pain persistence and illness behavior. A search for better drug targets is needed, but pharmacological treatment alone will not be sufficient; interdisciplinary management combining pharmacologic and non-pharmacologic approaches, including physical, cognitive behavioral, meditative, and occupational therapies, needs to be considered for chronic pain treatment ^88– 93^.

## Abbreviations

ATP, adenosine triphosphate; BDNF, brain-derived neurotrophic factor; CNS, central nervous system; CpG, cytosine–phosphate–guanine dinucleotide; CSF1, colony-stimulating factor 1; CXCR, chemokine CXC receptor; DMR, differentially methylated region; DRG, dorsal root ganglion; GABA, γ-aminobutyric acid; GABA _A_ receptor, γ-aminobutyric acid A receptor; IL, interleukin; Kv, voltage-gated potassium channel; lncRNA, long non-coding RNA; MCP-3, monocyte chemotactic protein-3; mGluR, metabotropic glutamate receptor; miRNA, microRNA; MOR, µ-opioid receptor; Nav, voltage-gated sodium channel; ncRNA, non-coding RNA; PPAR-γ, peroxisome proliferator-activated receptor-γ; ScNI, sciatic nerve injury; SNI, spinal nerve injury; TNF-α, tumor necrosis factor-α; TRPA1, transient receptor potential ankyrin 1; TSP-1, thrombospondin 1.
